# SPECT-CT metabolic and morphological study of 2 types of cemented hip stem prostheses in primary total hip arthroplasty patients: A protocol for a randomized controlled clinical trial (SPECT-PROTMA)

**DOI:** 10.1097/MD.0000000000028299

**Published:** 2021-12-30

**Authors:** Daniel Rodríguez, Thiago Carnaval, Marcos del Carmen, Azahara Palomar-Muñoz, Montserrat Cortés-Romera, José-Luis Agulló, Sebastián Videla

**Affiliations:** a Orthopedic Surgery and Traumatology Department, Bellvitge University Hospital, L’Hospitalet de Llobregat, Barcelona, Spain; b Clinical Research Support Unit (HUB-IDIBELL: Bellvitge University Hospital & Bellvitge Biomedical Research Institute), Clinical Pharmacology Department, Bellvitge University Hospital, L’Hospitalet de Llobregat, Barcelona, Spain; c Nuclear Medicine Department, Bellvitge University Hospital, L’Hospitalet de Llobregat, Barcelona, Spain; d Pharmacology Unit, Department of Pathology and Experimental Therapeutics, School of Medicine and Health Sciences, IDIBELL, University of Barcelona, L’Hospitalet de Llobregat, Barcelona, Spain.

**Keywords:** CT scan, French paradox, hip arthroplasty, metabolic changes, morphological changes, SPECT, thick-layer

## Abstract

**Background:**

Cemented hip arthroplasty requires applying a layer of polymethylmethacrylate (cement) in the space between the bone and the prosthetic stem. This can be achieved using 2 techniques: the thick-layer technique (requires a layer of at least 2 mm to surround an undersized prosthetic stem), and the thin-layer technique (requires a thin layer of cement, so that the prosthetic stem fills the femoral medullary canal). Both approaches have excellent long-term clinical and radiological outcomes, although an implant's insertion into the bone generates inevitable bone mass and bone metabolic changes around it. Combination of single photon emission computed tomography and computed tomography scan (SPECT-CT) imaging combines the single photon emission computed tomography's ability to provide detailed bone metabolism assessment with the computed tomography scan's capacity to provide a meticulous anatomical study.

**Methods:**

This is a single center, open label, randomized clinical trial, performed in the premises of the Bellvitge University Hospital. Participants will be randomly assigned to the Thick-layer technique group (Exeter V40 Cemented Femoral Stem) or to the French paradox technique group (Müller Straight Stem). All participants will have a SPECT-CT scan study at 3, 6, 12, and 24 months after the surgery.

**Discussion:**

Surgical distress itself and the implant's insertion into the bone may cause microvascular changes that alter periprosthetic bone mass and bone metabolism. To the best of our knowledge, there are no studies using SPECT-CT to compare bone metabolism evolution in the postoperative period between these 2 surgical cementation techniques. We aim to provide information in this regard that could help decision making in complicated implant cases and, maybe, pave the way for larger, and methodologically improved studies.

**Trial registration:**

NCT05010733 (August 18, 2021).

## 1 Introduction

### 1.1 Background and rationale

Since its introduction by John Charnley in 1959, total hip arthroplasty (THA) has been one of the most successful techniques in orthopedic surgery. Total hip replacement has progressively grown, reaching figures of a million arthroplasties implanted annually worldwide.^[1]^

Technically, the components of a total hip arthroplasty, which consist of an acetabular cup and of a femoral stem, can be attached to the bone through a cemented technique or a non-cemented technique. On the cemented hip arthroplasty, attachment of the prosthesis to the bone is done by using a cement (polymethylmethacrylate) that immediately stabilizes the implanted component. On the non-cemented hip arthroplasty, the prosthetic component is attached to the bone primarily under pressure (for primary stability) and, over time, osteointegration (a direct, structural, and functional connection between living bone and the prosthetic surface) is established.

Regarding the indication of each technique, some studies have shown good long-term results for both, and, in many cases, it is up to the surgeon in charge to decide which one to use. However, in situations of poor bone “stock”, such as elderly and/or osteoporotic patients, the cemented hip arthroplasty seems to be the best choice.^[2]^

Since the beginning in the early 1960s, surgical techniques and prosthetic designs have evolved remarkably. Considering the prosthetic's femoral stem design, and the cement layer that must surround it and attach it to the bone, there are 2 clearly different techniques^[3]^:

- **The thick-layer cement technique:** a cement layer of at least 2 millimeters (mm) surrounds a small enough (undersized) prosthetic stem to allow this layer of cement to fit between the femoral medullary canal and the prosthetic's femoral stem.
- **The thin-layer cement technique (also known as “The French Paradox**”**):** introduced in France in the early 1970s, this technique states that the prosthetic's femoral stem should “fill”, as much as possible, the femoral medullary canal, only requiring a thin layer of cement.

Both techniques have shown excellent long-term clinical and radiological outcomes. For this reason, the Orthopedic Surgery and Traumatology Department (OSTD) at the Bellvitge University Hospital uses both types of cemented prostheses (the Exeter V40 cemented femoral stem, for the thick-layer cement technique, and Müller Straight Stem, for the thin-layer cement technique).

Nevertheless, an implant's insertion into the bone (in this case, the femur) generates bone mass and bone metabolism changes around the implant. Monitoring these metabolic changes, and, therefore, the peri-prosthetic bone reaction, is of great importance when evaluating the results of each prosthetic model. There are many methods for peri-prosthetic bone changes monitoring, such as plain radiography, computed tomography (CT) scan, magnetic resonance imaging, dual energy radiography absorption and single photon emission computed tomography (SPECT). SPECT with technetium 99-labeled methyl-diphosphonates (MDP) is a useful tool for bone metabolism assessment^[4]^ and, although this technique may show insufficient anatomical details, its combination with CT scan (SPECT-CT) provides a solution and allows a combined metabolic and morphological study.^[5]^

To our knowledge, there are no studies comparing bone metabolism evolution in the postoperative period between these 2 approaches (thick-layer cement vs “French paradox”) with SPECT-CT. The aim of this work is to provide information on bone metabolic activity in the postoperative period of these 2 femoral stem models and to determine whether there are differences between them.

### 1.2 Explanation for the choice of comparators

We will compare the 2 types of cemented stem prostheses used for THA in our clinical practice: the Exeter V40 cemented femoral stem [Stryker Orthopaedics, Mahwah, NJ], for the thick-layer cement technique, and Müller straight stem [Zimmer, Winterthur, Switzerland], for the “French paradox” technique.

### 1.3 Objectives

The primary and secondary objectives are summarized in Table 1.

**Table 1:** Summarized study objectives.

Primary objectives
To describe the bone metabolic activity in cemented stem prostheses.
To describe the bone morphology in cemented stem prostheses.
Secondary objectives
To estimate the alignment angle.
To estimate the axial displacement degree.
To classify radiographically the prostheses’ risk of loosening according to the Harris Radiographic Classification of Loosening Risk (see Table S5, Supplemental Digital Content).
To estimate the clinical outcomes from THA according to the Harris Hip Score (see Table S1, Supplemental Digital Content).
To estimate the clinical outcomes from THA according to the Merle d’Aubigné Score (see Table S2, Supplemental Digital Content).
To estimate the clinical outcomes from THA according to the WOMAC Index (see Table S3, Supplemental Digital Content).
To estimate quality of life according to the EuroQoL-5D-3L© questionnaire (see Table S4, Supplemental Digital Content).

### 1.4 Trial design

This is a single center, randomized, controlled, pilot clinical trial in patients who undergo total hip arthroplasty surgery with implantation of a cemented prosthetic.

Those patients who accept to participate will have a plain radiography and a SPECT-CT, at 3, 6, 12, and 24 months of the intervention. These SPECT-CTs will be done in addition to the usual clinical practice complementary imaging exams and will require the administration of the ^99m^Technetium- methylene diphosphonate (^99m^Tc-MDP) radiopharmaceutical.

Since this is a study based on usual clinical practice, collection of existing information from medical records, and prospective evaluation of the SPECT-CTs, no significant risk is expected in advance for the patients enrolled. Besides, given the low doses of ^99m^Tc-MDP administered for this type of scan,^[6]^ radiation-induced adverse effects are extremely rare, especially in those over 60 years of age, which is the age-range of this study.^[7]^ However, an insurance cover will be hired, should any unforeseen problems arise because of the study.

The postoperative SPECT-CT follow-up results will be explained to each patient. After the 24-month SPECT-CT, the patients’ participation in the study will be terminated and usual clinical practice follow-up shall be provided as for the general population.

This study was registered in the ClinicalTrials.gov Protocol Registration and Results System (Identifier: NCT05010733, August 18, 2021).

## 2 Methods: participants, interventions, and outcomes

### 2.1 Study setting

This is a single-center clinical trial. Therefore, this study will be entirely carried out in Bellvitge University Hospital (L’Hospitalet de Llobregat, Barcelona, Spain) and will be coordinated by the OSTD. This is a fully equipped tertiary hospital.

### 2.2 Eligibility criteria

#### 2.2.1 Inclusion criteria

Patients ≥18 years-old, of both genders, undergoing a primary total hip arthroplasty surgery with implantation of a cemented prosthetic (Exeter V40 cemented femoral stem or Müller straight stem), with a diagnosis of hip osteoarthritis, operated by the OSTD at Bellvitge University Hospital during the 2 years after protocol's approval by the ethical committee, who have signed a written informed consent.

#### 2.2.2 Exclusion criteria

Patients allergic to ^99m^Tc-MDP radiopharmaceutical; with claustrophobia; with a background of an active septic process; a postoperative septic complication; periprosthetic fracture or misalignment of the prosthetic component; or those who have a total hip prosthetic implanted due to a sub capital femoral fracture.

### 2.3 Interventions

Patients will be randomized for 2 types of femoral cemented stem prostheses: the Exeter V40 cemented femoral stem [Stryker Orthopedics, Mahwah, NJ] for the thick-layer cement technique, and Müller straight stem [Zimmer, Winterthur, Switzerland] for the “French paradox” technique. These cemented stem prostheses used for THA are the ones used in our clinical practice.

As aforementioned, the study's main goal is to assess postoperative bone metabolism and bone morphology in patients who underwent THA. Therefore, SPECT-CTs will be performed. ^99^Tc-MDP SPECT-CTs will be performed as an additional complementary imaging test at 3, 6, 12, and 24 months after the surgical procedure. The acquisition field will be from the pelvis to the distal third of the femur, including the distal end of the prosthesis.

The SPECT-CTs study will combine bone metabolic activity and morphological assessment through the acquisition of SPECT-CT hybrid tomographic scans. The SPECT-CT will be performed with the *Discovery NM/CT 670* scan (*GE Healthcare, Waukesha, WI*). It has a dual-detector, free-geometry integrated nuclear imaging camera and a 16-head CT configuration.

Images’ acquisition will be performed as follows:

- Early phase: Planar dynamic images acquisition in vascular phase, injecting under the camera, in anterior and posterior projections.^[8]^ Acquisition parameters are Matrix 64x64, 2 seconds fm/60’.
- Immediately afterwards, a SPECT acquisition will be performed at an early stage in continuous “Step and shoot” mode, 7” fm/60 images per head (3° of rotation). Total acquisition: 7 minutes, Matrix 128x128, Zoom x1. No CT acquisition will be made.
- Late phase: Planar static images acquisition in anterior and posterior projection, 256x256 matrix, Zoom x1, for a period of 10 minutes. SPECT/TC: “Step and shoot” mode, 2” fm/60 images per head (3° of rotation). CT 120 kV, modulated mA (80-180) cuts 1.25 mm, matrix 512x512.

Image reconstruction uses Matrix 512x512, OSEM (*Volumetrix MI Evolution* for Bone, *GE Healthcare*) 128x128 and 4.4 mm pixel on each axis. OSEM iterative reconstruction (number of iterations 4, maximum of OSEM subsets 10). Periprosthetic activity quantification will be performed by applying regions of interest (ROIs) dividing the areas into segments, in both the acetabular and femoral components.^[9]^

Since the cement layer progressively attaches to the bone, a longitudinal evaluation must be performed. Risk of prosthetic loosening will be assessed according to the Harris Radiographic Loosening Risk Classification (Table S5, Supplemental Digital Content).[^10^,^11^]

Plain Radiographies will be assessed separately by 2 different physicians within the study team. In case of any discrepancies between their assessments, both physicians will discuss the plain radiographies until reaching a consensus. This consensus will be adopted as the final assessment.

Clinical outcomes of THA will be assessed by comparing patients’ pre- and postoperative answers to Harris Clinical Assessment scale (see Table S1, Supplemental Digital Content),[^12^,^13^] to Merle d’Aubigné Clinical Assessment Scale (see Table S2, Supplemental Digital Content),[^12^,^14^] and to Western Ontario and McMaster Universities Arthritis (WOMAC's Index) (Western Ontario and McMaster Universities Arthritis) (see Table S3, Supplemental Digital Content).^[15]^ Results in terms of quality of life will be assessed by comparing patients’ pre- and postoperative answers to the EuroQoL-5D-3L© questionnaire (see Table S4, Supplemental Digital Content).^[16]^

### 2.4 Criteria for discontinuing or modifying allocated interventions

Given the characteristics of this clinical trial, once the prostheses have been placed, the assigned interventions cannot be modified.

On the other hand, since the SPECT-CT scans will be performed to all enrolled patients, no modifications are anticipated. Participants may voluntarily discontinue their participation in the clinical trial for any reason, at any time. The investigator may also decide at any time during the trial, to temporarily interrupt or permanently discontinue the patients’ participation in the clinical trial if it is deemed that continuation would be detrimental to, or not in the best interest of the participant.

Similarly, the ethics committee or authorized regulatory authority can decide to halt or prematurely terminate the trial when new information becomes available whereby the rights, safety and well-being of trial participants can no longer be assured, when the integrity of the trial has been compromised, or when the scientific value of the trial has become obsolete and/or unjustifiable.

### 2.5 Strategies to improve adherence to intervention

As above mentioned, once the prostheses have been placed, the assigned interventions cannot be modified.

A team member will be responsible for programming and calling the patients in order to perform all planned control visits according to the protocol, scheduling the SPECT-CT and the planned questionnaires: Harris Hip Score, Merle d’Aubigné Score, WOMAC Index and EuroQoL-5D-3L©. Besides, a text message/phone call/email will be sent to the patients reminding them of the control visits 24 hour in advance.

### 2.6 Relevant concomitant care permitted or prohibited during the trial

Since in our hospital only 2 types of femoral cemented stem prostheses are available (Exeter V40 cemented femoral stem and Müller straight stem), which will be used in this clinical trial, it is not expected to use other femoral cemented stem prosthesis. The postoperative care (inpatient and outpatient) will be performed according to our clinical practice.

### 2.7 Outcomes

Study primary and secondary outcomes are summarized in Table 2.

**Table 2:** Summarized primary and secondary study outcomes.

Primary outcomes
Bone metabolic activity: number of counts per image set (median and its 95% CI) in the SPECT study. *Note: Counts per image set are a measure unit defined as the number of photons captured by the gamma camera, which represent the radiopharmaceutical uptake.* *SPECT will be performed at 3, 6, 12, and 24 mo of the intervention.*
Bone morphology: Alignment angles (median and its 95% CI) in the plain radiography and in the CT scan study. *Note: Alignment angles will be measured in degrees using the anatomical axis of the femur as a reference. Stem deviations from the femoral anatomical axis will be classified as “Varus” (if the tip of the stem is situated laterally to the femoral anatomical axis) or as “Valgus” (if the tip of the stem is situated medially to the femoral anatomical axis).* *Plain radiography will be performed at baseline and at 3, 6, 12, and 24 mo of the intervention.* *CT Scan will be performed at 3, 6, 12, and 24 mo of the intervention.*
Secondary outcomes
Axial displacement (median and its 95% CI) in the plain radiography and in the CT scan study. *Note: Axial displacement will be measured (in millimeters) using the inter-teardrop line as a reference. In the CT Scan study, this inter-teardrop line will be measured on the coronal plane.* *Axial displacement will be assessed at 3, 6, 12, and 24 mo of the intervention.*
Prosthesis loosening risk: number (and proportion) of patients classified as “possibly loose”, “probably loose”, and “definitely loose”, according to the Harris Radiographic Classification of Loosening Risk. *Note: Possibly Loose: Presence of a radiolucent line of 50% to 99% of the cement-bone boundary; Probably Loose: Radiolucency line at 100% of cement-bone boundary; Definitely Loose: Stem migration or a fracture of the cement mantle.*
Clinical outcomes: Harris Hip Score (median and its 95% CI). *Note: Being a score of 100 points the best possible result, and a score of 0 the worst possible result.*
Clinical outcomes: Merle d’Aubigné Score (median and its 95% CI) *Note: Clinical grades (Very good, Good, Medium, Fair, Poor) are given by the scores of Pain and Walking ability and adjusted down 1 to 2 grades, depending on the mobility score.*
Clinical outcomes: WOMAC Index Score (median and its 95% CI) *Note: Being a score of 96 the best possible result, and a score of 0 the worst possible result.*
QoL: EuroQoL-5D-3L© questionnaire score (median and its 95% CI). *Note: Since results are coded from 1 to 3 for each dimension, “11111” is the best possible result and “33333” is the worst possible result.*

#### 2.7.1 Study procedure

Participants will be screened among patients on the waiting list for THA. The screening will be carried out following the waiting list's order (i.e., the first patient on the waiting list will be contacted first, and so on, until the last patient on the waiting list is contacted or until the planned sample size has been reached, whichever comes first). Patients will be contacted by phone by a study team member, who will explain the study to them and answer any questions that may arise. If the patient accepts to participate, a screening visit will be scheduled.

On the day of “Visit 1”, the Study Information Sheet will be delivered to the patient and any other doubts that may arise will be answered. If the patient accepts to participate, he/she will be asked to sign the Informed Consent, a *sine qua non* condition for he/she to participate in the trial. Hereafter, all the information mentioned on Table 3 will be gathered and the complementary exams will be scheduled.

**Table 3:** Participant timeline.

	Visit 1	Visit 2	Visit 3	Visit 4	Visit 5	Visit 6	Visit 7
	Patient screening	Day of surgery	Hospital discharge	3 (±1 wk) mo of the surgery	6 (±2 wks) mo of the surgery	12 (±1) mo of the surgery	24 (±1) mo of the surgery
Obtaining informed consent	✓						
Eligibility criteria assessment	✓						
Demographic data	✓						
General examination (1)	✓	✓	✓	✓			
General laboratory blood tests (2)	✓		✓	✓	✓	✓	✓
Randomization		✓					
SPECT-CT				✓	✓	✓	✓
Plain radiography	✓			✓	✓	✓	✓
Clinical outcomes: Harris Hip Score, Merle d’Aubigné Score and WOMAC index score	✓			✓	✓	✓	✓
QoL questionnaire	✓			✓	✓	✓	✓
Safety: adverse events	✓	✓	✓	✓	✓	✓	✓
Concomitant treatment recording				✓	✓	✓	✓

The institutional review board (IRB) approved the protocol on May 20, 2021 (code ICPS033/20). We estimate that we will need approximately 6 months to identify the study population, establish the surgical indication, include them on the waiting list and perform the surgery. The study will be carried out between 2021 and 2024.

By the moment of submitting this manuscript, participant recruitment (patients’ screening visit) was still in progress.

### 2.8 Sample size

This pilot clinical trial is an exploratory study by nature. To our knowledge, there are no studies comparing bone metabolism evolution in the postoperative period of these 2 types of femoral stem prostheses with SPECT-CT. We have not proceeded to a formal sample size calculation. A total of 16 patients will be randomized (8 by group) in this clinical trial. This sample size is feasible and achievable with the available budget.

### 2.9 Recruitment

As abovementioned, patients will be screened among those on the waiting list for THA. First, when contacted by the principal investigator (or whoever he assigns), a detailed explanation of the study will be provided, and patients will be asked whether they want to participate and whether they intend to commit with all the scheduled visits. Likewise, on visit 1, the study will be explained in detail once again (emphasizing that the surgical indication will not differ from usual clinical practice and that the SPECT-CTs will be performed as an additional complementary imaging test at 3, 6, 12, and 24 months after the surgical procedure), and their commitment to assist to all scheduled visits will be asked once more. Posteriorly, the patient will be once again asked if he/she wants to participate in the trial, and, if positive, he/she will be requested to sign the written informed consent. During the study, a text message/phone call/email will be sent to the patients reminding them of the control visits 24 hours in advance. Besides, patients will be able to contact the principal investigator by phone at any time (the phone number will be provided in the Patient Information Sheet).

### 2.10 Assignment of interventions

#### 2.10.1 Allocation

##### 2.10.1.1 Sequence generation, concealment mechanism, and implementation

The principal investigator (DR) will oversee the screening procedure (i.e., contacting with patients and performing Visit 1). The randomization process will be centralized electronically using the e-CRF itself (research electronic data capture software [REDCap] platform) the day before the surgery. The randomization list will be computer-generated in blocks of 4. Patients will be assigned to the study groups at the time of enrollment on the study.

The randomization to the type of cemented stem prosthesis studied will be blinded to the participant and the surgeon team until the day of the surgery.

#### 2.10.2 Blinding (masking)

After assignment to interventions (Exeter V40 cemented femoral stem or Müller straight stem), both participant and surgical team will be aware of the type of cemented stem prosthesis allocated. Since after randomization this becomes an open-label study, emergency unblinding does not apply. Data analysts will be blinded.

Personal patient data will be coded and dissociated, so that the patient to whom they correspond is not recognizable. Consecutive numbers will be assigned as they are enrolled in the study, and these numbers (or codes) will be used in the electronic case report form (eCRF), rather than personal data.

### 2.11 Data collection plan

All data will be gathered from electronic medical records during the trial. All patients on the waiting list for total hip arthroplasty surgery during the study period will be screened and enrolled, if they meet the inclusion criteria and accept to participate, until reaching a total of 16 patients.

The study team will gather information on demographic characteristics, such as age and gender; body mass index; pathological background, particularly diabetes mellitus, hypertension, renal failure and vascular disease; preoperative anesthetic risk assessment (i.e., American Society of Anesthesiologists’ classification scale of overall physical health); clinical diagnosis that justify the surgical procedure indication; surgical approach; type of cemented stem prosthetic and its cementation; treatment with bone density altering drugs, such as corticosteroids or bisphosphonates, prior to the surgery; date of the intervention; date of hospital discharge; postoperative complications; pre- and postoperative clinical and radiographic assessment scales (i.e., the Harris and Merle d’Aubigne Scores, the WOMAC Index, the EuroQoL-5D-3L© questionnaire and the Harris Radiographic Classification of Loosening Risk).

The Harris Hip Score considers 4 clinical domains: pain, function, range of motion, and absence of deformity. Pain adds a maximum of 44 points; function, 47 points; range of motion, 5 points; and absence of deformity, 4 points. The maximum score is 100 points (best possible result), and the minimum is 0 (worst possible result)[^12^,^13^] (see Table S1, Supplemental Digital Content).

The Merle d’Aubigné Score considers pain, walking, and mobility. It is classified as “Very good”, “good”, “average”, “reasonable” and “poor”, according to pain and walking scores, and adjusted down 1 to 2 grades, according to the mobility score[^12^,^14^] (see Table S2, Supplemental Digital Content).

The WOMAC Index considers 3 different domains: pain (5 questions), stiffness (2 questions), and function (17 questions). The combined scores range from 0 to 96, being 96 the best possible result and 0 the worst possible result^[15]^ (see Table S3, Supplemental Digital Content).

The EuroQoL-5D-3L© questionnaire (EQ-5D-3L) was introduced in 1990. It is one of the most widely used instruments for measuring health-related quality of life. The EQ-5D-3L essentially consists of 2 pages: the EQ-5D descriptive system and the EQ-5D visual analogue scale (EQ VAS). The EQ-5D-3L descriptive system comprises the following 5 dimensions, each describing a different aspect of health: mobility, self-care, usual activities, pain/discomfort, and anxiety/depression. Each dimension has 3 levels: no problems, some problems, extreme problems (labelled 1-3). The respondent is asked to indicate his/her health state by checking the box against the most appropriate statement in each of the 5 dimensions.^[13]^ The EQ VAS records the respondent's self-rated health on a vertical VAS where the endpoints are labelled “The best health you can imagine” and “The worst health you can imagine”. This information can be used as a quantitative measure of health outcome as judged by the individual respondents^[16]^ (see Table S4, Supplemental Digital Content).

Permission for EuroQoL-5D-3L© questionnaire's use on this trial and for its reproduction on future related publications has been granted by the EuroQoL Office.

Harris radiographic classification of loosening risk is based on defined radiographic criteria and classifies the risk of stem loosening. The presence of a radiolucent line of 50% to 100% of the cement-bone boundary is defined as “possibly loose”. Radiolucency at 100% of this limit is classified as “probably loose”. Stem migration or a fracture of the cement mantle is classified as “definitely loose”. The more progressive the radiological lines, the more likely it is that there will be a prosthetic loosening[^10^,^11^] (see Table S5, Supplemental Digital Content).

SPECT-CT results will be recorded in the eCRF. Measurements on the prosthetic stem area will be performed considering 12 standard zones. SPECT results will be expressed as “counts per image set” (i.e., photons that represent the radiopharmaceutical uptake by the gamma camera) and as Standardized Uptake Values (SUVs) from each predetermined study areas.[^8^,^17^]

Quantification of periprosthetic activity will be performed by applying regions of interest, segmenting the areas in both the acetabular and femoral components. CT-scan results will provide data on bone density. The “comparative ratios” between the predetermined study area and its homologous contralateral counterpart will also be obtained. Early phase measurements will provide soft tissues’ activity information, and late phase measurements will provide information on bone formation.

An eCRF based on the REDCap platform (REDCap Consortium), has been created ad hoc for this study in coordination with the Biostatistics Unit of the IDIBELL and does not collect data that allows patient identification. Once the study is completed, the information collected will be transcribed into an anonymized ad hoc created database, which will be used for further statistical analysis.

### 2.12 Plans to promote participant retention and complete follow-up

Participants’ follow-up will be the same as for other patients not enrolled in this trial, except for the SPECT-CT scan that will be performed on post-operative months 3, 6, 12, and 24 after surgery. In other words, the Participants’ follow-up is the same to our clinical practice, except for SPECT-CT. This additional complementary imaging test will be carried out within the scope of the Bellvitge University Hospital, on the programmed clinical follow-up visit day and will not incur on extra expenses of any kind for the patients.

In each face-to-face medical follow-up visit, the clinical investigator in charge will remind the patient of the importance of correctly following the study and will encourage them to carry on in the clinical trial.

Protocol deviations will be documented and explained in detail by the investigators team (the sponsor is the principal investigator of this team). In the event of a “serious” protocol violation, the monitoring team will record all protocol breaches/deviations. The sponsor will review all protocol deviations and assess whether any of them represent a “serious” violation according to Good Clinical Practice guidelines. The sponsor will inform the IRB of any protocol breach/deviation that could impact on patient safety and on data integrity.

### 2.13 Data management

An electronic case report form (e-CRF), based on REDCap platform (REDCap Consortium), will be created ad hoc for this study in coordination with the Biostatistics Unit of the IDIBELL. It does not collect data that allows patient identification.

Before closing the database for analysis, the data manager and the principal investigator will check the completeness and accuracy of the recorded data. We do not expect confounding factors to influence the results, since this is primarily a descriptive study.

### 2.14 Statistical methods for analyzing primary and secondary outcomes

Baseline characteristics, type of surgery and its indication will be described using standard descriptive methods, and a descriptive and exploratory comparative analysis between both study groups will be carried out. The results will be expressed as means and standard deviation, median (maximum and minimum values) for the quantitative variables. Categorical variables will be expressed with the absolute and relative frequencies of each category.

Primary outcomes will be analyzed by determining the median (and its 95% confidence interval [95% CI]) of image-counts in the SPECT (to assess whether there are bone metabolic differences between the 2 types of cemented stem prostheses); and determining the median (and its 95% CI) of the alignment angles of the 2 types of cemented stem prostheses in plain radiography and CT scan (for morphological assessment).

Secondary outcomes will be analyzed by determining the median (and its 95% CI) of the axial displacement degree of the 2 types of cemented stem prostheses in plain radiography and CT scans; determining the median (and its 95% CI) of the interdigitation penetration range between the cement and the bone in the CT scans of both types of prostheses; calculating the proportion (and the absolute number) of patients classified as “possibly loose”, “probably loose” or “definitely loose” according to the radiographic fixation (loosening risk) of both types of prostheses in the plain radiography, according to the Harris Radiographic Classification of Loosening Risk[^10^,^11^]; determining the median Harris Hip Score (and its 95% CI),[^12^,^13^] pre- and postintervention, for both types of prostheses; determining the median Merle d’Aubigné Score (and its 95% CI),[^12^,^14^] pre- and postintervention, for both types of prostheses; determining the median WOMAC Index Score (and its 95% CI),^[15]^ pre- and postintervention, for both types of prostheses; determining the median EuroQoL-5D-3L© questionnaire score (and its 95% CI),^[16]^ pre- and postintervention, for the 2 types of prostheses.

Association studies will also be carried out. A 95% CI for the estimates shall be provided whenever it is possible.

Finally, the statistician who will perform the data analysis will be blinded regarding the study groups (Exeter V40 cemented femoral stem group or Müller Straight Stem group). R version 3.6.2 or higher for Windows (R Foundation for Statistical Computing) will be used for data processing and analysis.

### 2.15 Methods for any additional analyses

Additional analyses are not planned. Nevertheless, an exploratory multivariate regression model will be constructed and adjusted for potentially important confounding factors such as age, gender, clinical baseline characteristics.

### 2.16 Analysis population and missing data definition of analysis population relating to protocol nonadherence and any statistical methods to handle missing data

In the case of missing data, imputation will be made considering the treatment effects’ estimator is not biased and that an increase in type I error has been avoided. However, considering that the bone metabolic activity and bone morphology will be similar, and to ensure the trial's internal sensitivity in case of expecting a different evolution of one of the experimental groups, the last observation carried forward technique seems to be a conservative approach to the matter.

### 2.17 Oversight and monitoring

#### 2.17.1 Composition of the data monitoring committee, its role and reporting structure

A Data Safety and Monitoring Committee (DSMC) will be created ad hoc and will be composed by a medical doctor or pharmacist with expertise in pharmacovigilance, and by a medical doctor with THA expertise, both external to this protocol, and by a medical doctor indicated by the sponsor. The aim of this DSMC is to evaluate the safety of this clinical trial, mainly related to SPECT-TC. This DSMC will meet twice, when the patient number 10 completes the visit 3 and when the first 10 patients complete the first year of the study. The clinical trial could be stopped the DSMC deems necessary.

Since the intervention's known risks are minimal, and this is primarily an exploratory trial, trial monitoring will be carried out by members of the study team who will not be involved in the inclusion and follow-up of patients. The principal investigator will allow direct access to the trial data and corresponding data source, and to any other trial-related documents or materials to verify the accuracy and completeness of the data collected.

### 2.18 Description of any interim analyses and stopping guideline

It has not been planned to perform any interim analysis.

### 2.19 Adverse event reporting and harms

Adverse events recorded during the study will be coded according to the latest available version of the MedDRA dictionary and will be described using absolute and relative frequencies by study group, according to severity and its causal relation with treatment.

Serious adverse events will be described by study group and the 95% CI of the difference between both groups will be calculated.

Regarding the SPECT-CT, we do not expect any adverse reactions, since it is extremely rare for radio-induced secondary effects to appear (given the low doses of the radiopharmaceutical received^[6]^). Nonetheless, any adverse event that may occur during the trial will be assessed by the study team and, should any adverse reaction be related to the radiopharmaceutical administered, it would be collected in detail and notified to the Catalan Pharmacovigilance System.

### 2.20 Frequency and procedures for auditing trial conduct

The Investigator shall allow direct access to trial data and documents for monitoring, audits and/or inspections by competent regulatory or health authorities. As such, e-CRFs, source records and other trial-related documentation must be kept current, complete, and accurate at all times.

### 2.21 Ethics approval and consent to participate

The study protocol, version 2.1, was approved by the local IRB (Ethics and Clinical Investigation Committee of the Bellvitge University Hospital, code ICPS033/20, date of approval May 20, 2021). The list of local IRB members is available at: https://bellvitgehospital.cat/es/investiga-con-nosotros/ceic/composicion. Accessed on July 7, 2021.

This Trial will be conducted according to the criteria set by the Declaration of Helsinki (revised on WMA 64^th^ General Assembly, Fortaleza, Brazil, October, 2013), good clinical practice standards and applicable regulations. Although the surgical indication will not differ from usual clinical practice, SPECT-CTs are not usually performed for follow-up. In this study they will be performed as an additional complementary imaging test at 3, 6, 12, and 24 months from the intervention date. Since it requires the administration of the ^99m^Tc-MDP radiopharmaceutical and exposure to small doses of ionizing radiation, every patient that accepts to participate will be requested to sign a written informed consent prior to initiating any research activities. Furthermore, patients must be informed that their participation in this research is entirely voluntary, and that they can withdraw at any time, under no penalty risk whatsoever. Investigators’ participation in this study is free, voluntary, unpaid, and independent.

The level of confidentiality protection, in terms of personal data protection, as required by Spanish Law (Organic Law on Data Protection 3/2018), was also ensured.

Every patient that accepts to participate in the study will be assigned consecutive numbers as they are enrolled, and these numbers (or codes) will be used in the eCRF, instead of personal data. The data collected will be encoded, so that the patient to whom they correspond is not identified.

### 2.22 Plans for communicating important protocol amendments to relevant parties

Major protocol changes will be submitted for IRB approval and minor outcomes will be informed to the IRB. As per good clinical practice, trial participants will be informed of any significant changes during the trial.

### 2.23 Who will obtain informed consent?

Principal investigator (or another physician from the study team) will inform the screened patients about the study and ask them to sign the written informed consent in visit 1, whether the patient is interested in participating in the study. If he/she is interested to participate, the investigator of the study team will double-check the eligibility criteria before obtaining the signed written informed consent.

### 2.24 Additional consent provisions for collection and use of participant data and biological specimens

Currently, the study team does not intend to carry out any ancillary studies. If any future studies are planned to be carried out later using the anonymized stored data, a new protocol should be made, and a new IRB approval should be sought.

### 2.25 Confidentiality

The results from this clinical trial are confidential and may not be transferred to third parties in any form or manner without written permission from the Sponsor. All individuals involved in the clinical trial are bound to this confidentiality clause in line with the REGULATION (EU) 2016/679 OF THE EUROPEAN PARLIAMENT AND OF THE COUNCIL of April 27, 2016 on the protection of natural persons with regard to the processing of personal data and on the free movement of such data, as well as all other valid and applicable laws and regulations, such as the “*Ley Orgánica 3/2018, de 5 de diciembre, de Protección de Datos Personales y garantía de los derechos digitales*” [Personal Data Protection and Digital Rights Assurance Law]. Therefore, patient data will be pseudonymized.

Whilst obtaining a signature for the Written Informed Consent, the Investigator will request written permission from the patient to directly access his/her data. With this permission granted, the patient's data may be examined, analyzed, verified, and reproduced for the evaluation of the clinical trial.

Data will be anonymized, so that the corresponding patient cannot be identified. Patient data will also be dissociated. Patients will be assigned consecutive numbers as they are enrolled in the study, and these identification numbers (or codes) will be used in the e-CRF; the full name of the patient will not be included in the e-CRFs. The principal investigator of each center will keep an updated patient identification list containing the name, clinical history number and the patient's identification number (or code) for the clinical trial.

The study monitor may have access to the patient's identity and data related to the study monitoring procedures. Any person with direct access to the data (Regulatory Authorities, Trial Monitors, and auditors) will take all possible precautions to maintain the confidentiality of patient's identities.

It is the Investigator's responsibility to obtain a written informed consent from the study patients. It is the Trial Monitor's responsibility to make sure that each patient has given his/her written consent to allow this direct access.

The Investigator shall ensure that the documents provided to the Sponsor do not contain the patient's name or any identifiable data.

### 2.26 Declaration of interests

The authors declare no conflict of interests.

### 2.27 Availability of data and materials

The database management and statistical analysis will be carried out by the Biostatistics Unit of the IDIBELL. The data sets used and/or analyzed during the study will be available from the corresponding author upon reasonable request.

### 2.28 Ancillary and post-trial care

A specific insurance has been hired ad hoc, in case of any harm related to a patient's participation. Since this trial is based on our usual clinical practice (i.e., the prosthetic devices that will be used for this study are the same currently being used in our clinical practice), on the collection of existing information in the medical history, and on the prospective assessment of SPECT-CT scans, no significant risk is anticipated for the research subjects. In the area of diagnostic imaging, both in Radiodiagnosis and Nuclear Medicine, it is extremely rare for radio-induced secondary effects to appear, given the low doses received because of this type of test,^[6]^ especially to those over 60 years-old, which is the age-range expected for this study.^[7]^

### 2.29 Dissemination policy: trial results, authorship

The study findings will be submitted to a peer-reviewed journal for publication and presented at relevant national and international scientific meetings.

The authorship is based on the criteria according to International Committee of Medical Journal Editors http://www.icmje.org/recommendations/browse/roles-and-responsibilities/defining-the-role-of-authors-and-contributors.html. Accessed July 7, 2021.

### 2.30 Plans to give access to the full protocol, participant level-data and statistical code

The protocol is available on clinicaltrials.gov (NCT05010733). No public access to the patient dataset is planned to be given at this moment. Dr. Cristian Tebé, the Head of the Biostatistics Unit, will oversee the dataset and granting access to this information will be evaluated on a case-by-case basis and upon reasonable request by the interested part.

## 3 Discussion

The rationale for this trial is that the surgical steps taken prior to implant insertion and implant insertion itself may cause microvascular changes and distress that alter periprosthetic bone mass and metabolism. We intend to quantify these bone metabolic and morphological changes, as well as the prosthetics’ alignment angles, to assess whether there are differences between both types of prostheses. ^99^Tc-MDP SPECT is a useful tool for bone metabolism assessment,^[4]^ and when associated with the CT scan (SPECT-CT) allows a combined metabolic and morphological study.^[5]^

Additionally, images acquired from the contralateral hip area will allow comparison between the periprosthetic bone metabolism and the patient's regular hip bone metabolism (i.e., not submitted to surgical distress).

SPECT-PROTMA has limitations. The ideal design would be a double-blind, randomized clinical trial, which is not possible in our setting because of limited personnel (the study team responsible for selecting the patients is the same responsible for performing the surgery and for follow up). Besides, since this is an independent study, the project's budget is an important limitation. Hence, our sample size is small (16 participants is the maximum amount that we can enroll without an added radiopharmaceutical cost) and might be a drawback.

A priori, SPECT-PROTMA will not provide a direct benefit for the patients enrolled, since it is mostly an exploratory study. It will generate, though, knowledge regarding their bone metabolism status throughout the first 2 years after the surgery.

We believe the results from this trial could help design better follow-up guidelines for this kind of patients; might support decision making in complicated prosthetic implants’ cases (even in a scenario where a replacement surgery is mandatory); and might pave the way for larger, methodologically improved studies to assess long-term bone metabolism on uncomplicated THA cemented stem prostheses.

∗*Note: This protocol was written following the SPIRIT guideline*.^[18]^

### 3.1 Trial status

The current protocol version is 2.1 (April, 2021). This trial is currently on the screening phase (August, 2021).

### 3.2 Informed consent materials

Please refer to Supplementary Material 6, Supplemental Digital Content and Supplementary Material 7, Supplemental Digital Content.

### 3.3 Plans for collection, laboratory evaluation, and storage of biological specimens for genetic or molecular analysis in the current trial and for future use in ancillary studies, if applicable

Blood samples will be extracted following usual clinical practice guidelines, and in a presurgical and postsurgical follow-up setting. No biological samples will be stored for future use.

## Acknowledgments

*We thank* Bellvitge University Hospital, *IDIBELL and CERCA Program/Generalitat de Catalunya for institutional support.* We also would like to thank the support provided by Alejandro Badenes Romo (technician of the Nuclear Medicine Department of the Bellvitge University Hospital), Gabriel Reynés Llompart (physicist of the Medical Physics and Radioprotection Department of the Catalan Institute of Oncology, L’Hospitalet de Llobregat, Barcelona, Spain).

## Author contributions

All Authors have read and approved the manuscript.

**Conceptualization:** DR, TC, SV

**Data curation:** DR, SV

**Funding acquisition:** DR

**Investigation:** Marcos del Carmen, Azahara Palomar-Muñoz, Montserrat Cortés-Romera, José-Luis Agulló

**Methodology:** TC, SV

**Supervision:** DR, SV

**Writing – original draft:** DR, TC, SV

**Writing – review & editing:** DR, TC, SV

## Supplementary Material

Supplemental Digital Content

## Supplementary Material

Supplemental Digital Content

## Supplementary Material

Supplemental Digital Content

## Supplementary Material

Supplemental Digital Content

## Supplementary Material

Supplemental Digital Content

## Supplementary Material

Supplemental Digital Content

## Supplementary Material

Supplemental Digital Content
