# Supplementary material for: SPECT-CT metabolic and morphological study of 2 types of cemented hip stem prostheses in primary total hip arthroplasty patients: A protocol for a randomized controlled clinical trial (SPECT-PROTMA)
Source: Medicine (Baltimore). 2021 Dec 30;100(52):e28299. doi: 10.1097/MD.0000000000028299 (PMC8718198; doi:10.1097/MD.0000000000028299)
Supplement: Supplemental Digital Content [file medi-100-e28299-s001.docx]

**Supplementary Table 5: Harris’ radiographic loosening risk classification:**

**_____________________________________________________________________________________**

**1. Possibly loose:**

Presence of a radiolucent line ≥ 50% of the cement-bone boundary and <100%

**2. Probably loose:**

Presence of a radiolucent line that is 100% of the interface

**3. Definitely loose:**

Migration of the prosthetic component or presence of a fracture on the cement mantle
