# Supplementary material for: SPECT-CT metabolic and morphological study of 2 types of cemented hip stem prostheses in primary total hip arthroplasty patients: A protocol for a randomized controlled clinical trial (SPECT-PROTMA)
Source: Medicine (Baltimore). 2021 Dec 30;100(52):e28299. doi: 10.1097/MD.0000000000028299 (PMC8718198; doi:10.1097/MD.0000000000028299)
Supplement: Supplemental Digital Content [file medi-100-e28299-s002.doc]

**Supplementary Table 1:**

| **I. Pain** |  |
| --- | --- |
| 1. None or ignores it | 44 |
| 1. Slight, ocasional, no compromise in activities | 40 |
| 1. Mild pain, no effect on average activities, rarely moderate pain with unusual activity, may take aspirin | 30 |
| 1. Moderate pain, tolerable but makes concessions to pain. Some limitation of ordinary activity or work.May require occasional pain medicine stronger than aspirin | 20 |
| 1. Marked pain, serious limitations of activities | 10 |
| 1. Totally disabled, crippled, pain in bed, bedridden | 0 |
| **II. Function** |  |
| 1. **Gait** |  |
| 1. **Limp** |  |
| 1. None | 11 |
| 1. slight | 8 |
| 1. Moderate | 5 |
| 1. Severe | 0 |
| 1. **Support** |  |
| 1. None | 11 |
| 1. Cane for long walks | 7 |
| 1. Cane most of the time | 5 |
| 1. One crutch | 3 |
| 1. Two canes | 2 |
| 1. Two crutches | 0 |
| 1. Not able to walk | 0 |
| 1. **Activities** |  |
| 1. **Stairs** |  |
| 1. Normally without using any railing | 4 |
| 1. Normally using a railing | 2 |
| 1. In any manner | 1 |
| 1. Unable to use stairs | 0 |
| 1. **Shoes and Socks** |  |
| 1. With ease | 4 |
| 1. With difficulty | 2 |
| 1. Unable | 0 |
| 1. **Sitting** |  |
| 1. Sitting comfortably in ordinary chair for 1h | 5 |
| 1. Sitting on a high chair for one/half hour | 3 |
| 1. Unable to sit comfortably in any chair | 0 |
| 1. **Distance** |  |
| 1. Unlimited | 11 |
| 1. 6 blocks | 8 |
| 1. 2 or 3 blocks | 5 |
| 1. Indoors only | 2 |
| 1. Bed and chair | 0 |
| 1. **Enter Public Transportation** | 1 |
| **III. Absence of deformity points (4) are given if the patient demonstrates:** |  |
| 1. Less than 30° fixed flexion contracture | 1 |
| 1. Less than 10° fixed adduction | 1 |
| 1. Less than 10° fixed internal rotation in extension | 1 |
| 1. Limb-length discrepancy less than 3.2 cm | 1 |
| **IV. Range of motion (ROM): Index values are determined by multiplying the degrees of motion by the indices. The ROM score is given by adding the index values multiplying with the factor 0.05. Maximum score gives 5 points** |  |
| 1. Flexion: 0–45° × 1.0, 45–90° × 0.6, 90–110° × 0.3 |  |
| 1. Abduction: 0–15° × 0.8, 15–20° × 0.3, > 20° × 0 |  |
| 1. External rotation in extension: 0–15° × 0.4, >15° × 0 |  |
| 1. Internal rotation in extension: any × 0 |  |
| 1. Adduction: 0–15° × 0.2 |  |

**The Harris’ Hip Score Scale:** it consists of the domains Pain, Function, Deformity and Range of Motion (ROM), and gives a maximum of 100 points in the total score.
