# Supplementary material for: SPECT-CT metabolic and morphological study of 2 types of cemented hip stem prostheses in primary total hip arthroplasty patients: A protocol for a randomized controlled clinical trial (SPECT-PROTMA)
Source: Medicine (Baltimore). 2021 Dec 30;100(52):e28299. doi: 10.1097/MD.0000000000028299 (PMC8718198; doi:10.1097/MD.0000000000028299)
Supplement: Supplemental Digital Content [file medi-100-e28299-s003.docx]

**Supplementary Table 2:**

| **Score** | **Pain** | **Walking Capacity** | **Mobility** |
| --- | --- | --- | --- |
| 0 | Pain is intense and permanent | None | Ankylosis with bad position of the hip |
| 1 | Pain is severe, even at night | Only with crutches | No movement; pain or slight deformity |
| 2 | Pain is severe when walking; prevents any activity | Only with canes | Flexion under 40 degrees |
| 3 | Pain is tolerable with limited activity | With 1 cane, less than 1 h; very difficult | Flexion between 40 and 60 degrees |
| 4 | Pain is mild when walking; it disappears with rest | A long time with a cane; short time without cane and with limp | Flexion between 60 and 80 degrees; patient can reach his/her foot |
| 5 | Pain is mild and inconstant; normal activity | Without cane, but with slight limp | Flexion between 80 and 90 degrees; abduction of at least 15 degrees |
| 6 | No pain | Normal | Flexion more than 90 degrees; abduction to 30 degrees |

**Merle d’Aubigné-Postel Score**: The individual scores of Pain, Walking ability and Mobility are added together to give an overall numeric score. Clinical grades (Very good, Good, Medium, Fair, Poor) are given by the scores of Pain and Walking ability and adjusted down 1–2 grades, depending on the mobility score.
