# Supplementary material for: SPECT-CT metabolic and morphological study of 2 types of cemented hip stem prostheses in primary total hip arthroplasty patients: A protocol for a randomized controlled clinical trial (SPECT-PROTMA)
Source: Medicine (Baltimore). 2021 Dec 30;100(52):e28299. doi: 10.1097/MD.0000000000028299 (PMC8718198; doi:10.1097/MD.0000000000028299)
Supplement: Supplemental Digital Content [file medi-100-e28299-s004.docx]

**Supplementary Table 3:**

**_____________________________________________________________________________________**

**Pain**

- Walking on flat surface 0 1 2 3 4
- Going up or down stairs 0 1 2 3 4
- At night while in bed 0 1 2 3 4
- Sitting or lying down 0 1 2 3 4
- Standing upright 0 1 2 3 4

**Stiffness**

- Stiffness after first awakening in the morning 0 1 2 3 4
- Stiffness after sitting, lying or resting later in the day 0 1 2 3 4

**Physical Capacity – Level of difficulty when...**

- Going down stairs 0 1 2 3 4
- Going up stairs 0 1 2 3 4
- Rising from sitting 0 1 2 3 4
- Standing 0 1 2 3 4
- Bending to floor 0 1 2 3 4
- Walking on a flat surface 0 1 2 3 4
- Getting in/out of car 0 1 2 3 4
- Going shopping 0 1 2 3 4
- Putting on socks/stockings 0 1 2 3 4
- Rising from bed 0 1 2 3 4
- Taking off socks/stockings 0 1 2 3 4
- Lying in bed 0 1 2 3 4
- Getting in/out of bath 0 1 2 3 4
- Sitting 0 1 2 3 4
- Getting on/off toilet 0 1 2 3 4
- Performing heavy domestic duties* 0 1 2 3 4
- Performing heavy domestic duties** 0 1 2 3 4

**_____________________________________________________________________________________**

**The WOMAC Index**: it assesses 3 different dimensions: pain (5 questions), stiffness (2 questions), and function (17 questions). The combined scores range from 0 to 96, with 96 being the best possible result. It is rated as follows:

(0): No; (1): Mild; (2): Moderate; (3): Very; (4): Extremely

**e.g.,* mowing the lawn, lifting heavy grocery bags

***e.g.,* tidying a room, dusting, cooking
