# Supplementary material for: SPECT-CT metabolic and morphological study of 2 types of cemented hip stem prostheses in primary total hip arthroplasty patients: A protocol for a randomized controlled clinical trial (SPECT-PROTMA)
Source: Medicine (Baltimore). 2021 Dec 30;100(52):e28299. doi: 10.1097/MD.0000000000028299 (PMC8718198; doi:10.1097/MD.0000000000028299)
Supplement: Supplemental Digital Content [file medi-100-e28299-s005.docx]

**Supplementary Table 4:**

**The EuroQoL-5D-3L© Scale:**

**MOBILITY**

I have no problems in walking about O
I have some problems in walking about O
I am confined to bed O

**SELF-CARE**

I have no problems washing or dressing myself O
I have some problems washing or dressing myself O
I am unable to wash or dress myself O

**USUAL ACTIVITIES (*e.g., work, study, housework, family or leisure activities*)**

I have no problems doing my usual activities O
I have some problems doing my usual activities O
I am unable to do my usual activities O

**PAIN/DISCOMFORT**

I have no pain or discomfort O
I have moderate pain or discomfort O
I have extreme pain or discomfort O

**ANXIETY/DEPRESSION**

I am not anxious or depressed O
I am moderately anxious or depressed O
I am extremely anxious or depressed O

**The EQ-5D-3L Questionnaire:** it consists of a descriptive system that comprises the following 5 dimensions, each describing a different aspect of health: **MOBILITY**, **SELF-CARE**, **USUAL ACTIVITIES**, **PAIN**/**DISCOMFORT** and **ANXIETY**/**DEPRESSION**. Each dimension has 3 levels: no problems, some problems, extreme problems (labelled 1–3). The respondent is asked to indicate his/her health state by checking the box against the most appropriate statement in each of the five dimensions.

**The EuroQoL VAS Scale:**

- We would like to know how good or bad your health is TODAY.
- This scale is numbered from 0 to 100.
- 100 means the best health you can imagine. 0 means the worst health you can imagine.
- Mark an X on the scale to indicate how your health is TODAY.
- Now, please write the number you marked on the scale in the box below.

The best health you can imagine


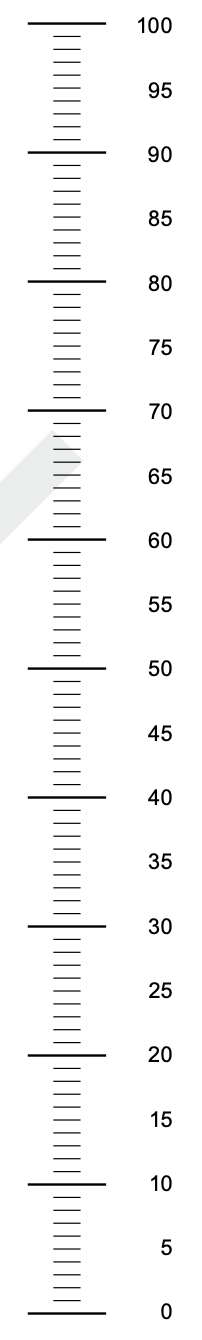


The EQ VAS records the respondent’s self-rated health on a vertical VAS where the endpoints are labelled ‘The best health you can imagine’ and ‘The worst health you can imagine’. This information can be used as a quantitative measure of health outcome as judged by the individual respondents.

Your Health Today =

The worst health you can imagine
