# Supplementary material for: SPECT-CT metabolic and morphological study of 2 types of cemented hip stem prostheses in primary total hip arthroplasty patients: A protocol for a randomized controlled clinical trial (SPECT-PROTMA)
Source: Medicine (Baltimore). 2021 Dec 30;100(52):e28299. doi: 10.1097/MD.0000000000028299 (PMC8718198; doi:10.1097/MD.0000000000028299)
Supplement: Supplemental Digital Content [file medi-100-e28299-s006.docx]

**Model consent form and other related documentation given to participants and authorized surrogates**

**HOJA DE CONSENTIMIENTO INFORMADO**

**TITULO DEL ESTUDIO:**

"ESTUDIO METABÓLICO Y MORFOLÓGICO DE DOS DISEÑOS DE TALLO CEMENTADO IMPLANTADOS EN CIRUGÍA PRIMARIA DE PRÓTESIS TOTAL DE CADERA".

**CÓDIGO DE PROTOCOLO:** HUB-COT-2020-01

**Yo**, _________________________________________________________________________________:

⎕ He leído la hoja de información que se me ha entregado sobre el estudio. He podido hacer preguntas sobre el estudio.

⎕ He recibido suficiente información sobre el estudio.

⎕ He hablado con el investigador Dr. Daniel Rodríguez Pérez.

⎕ Comprendo que mi participación es voluntaria.

⎕ Comprendo que puedo retirarme del estudio:

- Cuando quiera.
- Sin tener que dar explicaciones.
- Sin que ello repercuta en modo a mi atención médica.

Recibiré una copia firmada y con fecha de este documento de consentimiento informado. Doy libremente mi conformidad para participar en el estudio.

**Firma del/de la participante:**

**Fecha: ____/____/____**

**Firma del investigador:**

**Fecha: ____/____/____**
