# Supplementary material for: SPECT-CT metabolic and morphological study of 2 types of cemented hip stem prostheses in primary total hip arthroplasty patients: A protocol for a randomized controlled clinical trial (SPECT-PROTMA)
Source: Medicine (Baltimore). 2021 Dec 30;100(52):e28299. doi: 10.1097/MD.0000000000028299 (PMC8718198; doi:10.1097/MD.0000000000028299)
Supplement: Supplemental Digital Content [file medi-100-e28299-s007.docx]

**Model consent form and other related documentation given to participants and authorized surrogates**

**HOJA DE INFORMACIÓN AL PACIENTE**

**TÍTULO DEL ESTUDIO:**

"ESTUDIO METABÓLICO Y MORFOLÓGICO DE DOS DISEÑOS DE TALLO CEMENTADO IMPLANTADOS EN CIRUGÍA PRIMARIA DE PRÓTESIS TOTAL DE CADERA".

**CÓDIGO DEL ESTUDIO:** HUB-COT-2020-03

**PROMOTOR:** Dr. Daniel Rodríguez Pérez. Unidad de Cadera, Servicio de Cirugía Ortopédica y Traumatología, Hospital Universitario de Bellvitge

**INVESTIGADOR PRINCIPAL:** Dr. Daniel Rodríguez Pérez

**CENTRO:** Hospital Universitario de Bellvitge

**INTRODUCCIÓN:**

Nos dirigimos a usted para informarle sobre el estudio de investigación al que se le / la invita a participar. El estudio ha sido aprobado por el Comité de Ética del Hospital Universitario de Bellvitge. Nuestra intención es que usted reciba la información correcta y suficiente para que pueda decidir si acepta o no participar en este estudio. Por ello lea esta hoja informativa con atención y nosotros le aclararemos las dudas que puedan surgir. Además, puede consultar con las personas que considere oportuno.

**PARTICIPACIÓN VOLUNTARIA:**

Debe saber que su participación en este estudio es voluntaria y puede decidir no participar. Si decide participar, puede cambiar su decisión y retirar el consentimiento en cualquier momento, sin que por ello se altere la relación con su médico ni se produzca ningún perjuicio a su atención sanitaria.

**OBJETIVO DE LA ESTUDIO:**

Estudio metabólico y morfológico con SPECT / CT en el plazo de un año de 16 prótesis totales de cadera con tallo cimentada implantadas en la Unidad de Cadera del Servicio de Cirugía Ortopédica y Traumatología.

**DESCRIPCIÓN ESTUDIO:**

Visita habitual en consulta externa para valoración de coxartrosis. Se le realizará anamnesis, exploración física y evaluación radiológica. Si el paciente tiene indicación de prótesis cementada y cumple los criterios de inclusión, se le propondrá participar en el estudio. Una vez firmado el consentimiento informado de la intervención y del estudio, se procederá a evaluación con dos escalas de valoración clínica que rellenará el facultativo (Harris y Merle d’Aubigné) y dos que rellenará el propio paciente (WOMAC y EuroQoL-5D-3L©). El paciente entrará lista de espera, será programado para intervención quirúrgica y será operado. Tras procederse a la intervención quirúrgica, se solicitará una radiografía simple y una SPECT / CT a los 3 meses, a los 6 meses, al año y a los dos años de la intervención, así como la evaluación con escalas de valoración clínica citadas. Estas pruebas de imagen con SPECT/TC se añaden a las pruebas de imagen habituales y servirán para obtener datos de la actividad metabólica del fémur alrededor del tallo de la prótesis implantada. Los datos y su tratamiento serán exclusivamente para la realización este estudio y en ningún caso se utilizarán para otros fines.

En las visitas postoperatorias habituales, se informará al paciente de los resultados de las exploraciones complementarias.

**RIESGOS Y MOLESTIAS DERIVADAS DE LA PARTICIPACIÓN EN EL ESTUDIO:**

Hacia lo que respecta a la evaluación clínica. Respecto a las pruebas de imagen (SPECT/TC y radiología simple), se han de tener en cuenta las consideraciones de cualquier exploración con rayos X el riesgo potencial de radiación incluye una ligera elevación del riesgo de padecer cáncer al cabo de unos años. Teniendo en cuenta los aparatos de nueva generación y las técnicas actuales, este es un riesgo muy pequeño en el corto y en el largo plazo.

**ADVERTENCIA RELATIVA AL EMBARAZO:**

En caso de estar en situación de gestación previamente o durante el estudio, informará inmediatamente al investigador.

**Protección de datos personales:**

El promotor se compromete a que toda la información (personal y clínica) recogida de los pacientes se tratará de acuerdo con la Ley Orgánica 3/2018 de protección de Datos Personales y Garantía de los Derechos Digitales. Los datos recogidos para el estudio estarán identificados mediante un código, por lo que no incluya información que pueda identificarlo, y sólo su médico del estudio / colaboradores podrá relacionar estos datos con usted y con su historia clínica. Por lo tanto, su identidad no será revelada a ninguna persona salvo excepciones en caso de urgencia médica o requerimiento legal. El tratamiento, la comunicación y la cesión de los datos de carácter personal de todos los participantes se ajustarán a lo dispuesto en esta ley.

Desde el 25 de mayo de 2018 es de plena aplicación la nueva legislación en la UE sobre datos personales, en concreto el Reglamento (UE) 2016/679 del Parlamento Europeo y del Consejo de 27 de abril de 2016 Protección de Datos (RGPD). Por ello, es importante que conozca la siguiente información:

- Además de los derechos que ya conoce (acceso, modificación, oposición y cancelación de datos) ahora también puede limitar el tratamiento de datos que sean incorrectos, solicitar una copia o que se trasladen a un tercero (portabilidad) los datos que usted ha facilitado para el estudio. Para ejercitar sus derechos, se puede dirigir al investigador principal del estudio o al delegado de protección de datos de nuestra institución (FUNDACIÓN TIC SALUD SOCIAL: dpd@ticsalutsocial.cat). Le recordamos que los datos no se pueden eliminar, aunque deje de participar en el ensayo para garantizar la validez de la investigación y cumplir con los deberes legales y los requisitos de autorización de medicamentos. Asimismo, tiene derecho a dirigirse a la Autoridad Catalana de Protección de Datos si no quedara satisfecho.
- Tanto el Centro como el Promotor son responsables respectivamente del tratamiento de sus datos y se comprometen a cumplir con la normativa de protección de datos en vigor. Los datos recogidos para el estudio estarán identificados mediante un código, por lo que no se incluya información que pueda identificarlo, y sólo su médico del estudio / colaboradores podrá relacionar estos datos con usted y con la su historia clínica. Por lo tanto, su identidad no será revelada a ninguna otra persona excepto a las autoridades sanitarias, cuando así lo requieran o en casos de urgencia médica. Los Comités de Ética de la Investigación, los representantes de la autoridad sanitaria en materia de inspección y el personal autorizado por el Promotor, únicamente podrán acceder para comprobar los datos personales, los procedimientos del estudio clínico y el cumplimiento de las normas de buena práctica clínica (siempre manteniendo la confidencialidad de la información).
- El Investigador y el Promotor están obligados a conservar los datos recogidos para el estudio como mínimo hasta 25 años después de su finalización. Posteriormente, su información personal sólo se conservará por el centro para el cuidado de su salud y por el promotor para otros fines de investigación científica si usted hubiera otorgado su consentimiento para ello y si así lo permite la ley y requisitos éticos aplicables.

**OTRA INFORMACIÓN RELEVANTE:**

Debe saber que puede ser excluido del estudio si el promotor o los investigadores del estudio lo consideran oportuno, ya sea por motivos de seguridad, para cualquier evento adverso que se produzca o porque consideran que no está cumpliendo con los procedimientos establecidos. En cualquiera de los casos, usted recibirá una explicación adecuada del motivo que ha ocasionado su retirada del estudio.

Al firmar la hoja de consentimiento adjunta, se compromete a cumplir con los procedimientos del estudio que se le han expuesto.

**CONTACTO EN CASO DE DUDAS:**

Si durante su participación tiene alguna duda o necesita más información, póngase en contacto con el Dr. Daniel Rodríguez Pérez, del servicio de Cirugía Ortopédica y Traumatología (Unidad de Cirugía de Cadera).

**Teléfono:** 932607500 Ext. 7572
